# Physicochemical and major ion data for springs in the Black Forest National Park, Germany

**DOI:** 10.1016/j.dib.2020.105645

**Published:** 2020-04-30

**Authors:** Markus Merk, Nadine Goeppert, Nico Goldscheider

**Affiliations:** Institute of Applied Geosciences, Division of Hydrogeology, Karlsruhe Institute of Technology (KIT), Kaiserstr. 12, 76131 Karlsruhe, Germany

**Keywords:** Spring water sampling, Hydrochemical baseline study, Fractured sandstone aquifer, Organic carbon, Mountain hydrology, National Park

## Abstract

The dataset in this article consists of the general physicochemical parameters (temperature, pH, specific electrical conductivity, dissolved oxygen, redox potential, alkalinity) and concentrations of major ions (Ca^2+^, Mg^2+^, K^+^, Na^+^, Cl^-^, SO_4_^2-^, NO_3_^-^) of water samples collected at 19 springs and the surface stream in the water catchment area of the upper Schönmünz river in the Black Forest National Park, Germany. Data on concentrations of dissolved organic carbon (DOC), total organic carbon (TOC), spectral absorbance at different wavelengths and fluorescence as well as microbiological indicators (*E. coli*, total coliforms, enterococci) are also reported. Sampling was conducted during five field campaigns between spring 2016 and spring 2017.

Knowledge of the current physicochemical parameters and concentrations of dissolved organic and inorganic constituents provides a baseline to assess future changes and serves as a supplement to ongoing studies of the spring ecosystems. Understanding the specific processes influencing the water chemistry will aid in their effective protection.

For more details and further discussion on this dataset, the reader is referred to the associated research article “Processes controlling spatial and temporal dynamics of spring water chemistry in the Black Forest National Park” [1]

**Specifications Table**

**Table:** 

**Subject**	Environmental Science (General)
**Specific subject area**	Spring water chemistry
**Type of data**	Table
**How data were acquired**	Mg^2+^, K^+^, Na^+^: ion chromatography, ICS-1100, Thermo Fisher Scientific Ca^2+^: flame AAS, Perkin-Elmer 3030 B Cl^-^, SO_4_^2-^, NO_3_^-^: ion chromatography, ICS-2100, Thermo Fisher Scientific TOC, DOC: oxidative combustion, Vario TOC cube, Elementar Analysesysteme GmbH absorbance spectra, fluorescence excitation-emission matrices: Aqualog, Horiba Scientific Silica: classical molybdenum blue method, Photolab 6600 UV-Vis, WTW Al: Spectroquant aluminum test, Merck total iron: Aquaquant iron test, Merck Coliforms/*E. coli*: Idexx Colisure, Quanti-Tray/2000 Enterococci: Idexx Enterolert, Quanti-Tray/2000
**Data format**	Raw (water chemistry data) Processed [Humification index (HIX) and biological index (BIX), derived from fluorescence measurements] Shapefile (all selected sampling locations)
**Parameters for data collection**	Water samples were collected at 19 springs and the surface stream during five different field campaigns in different seasons. Selection of springs was based on stratigraphy, spring type as well as flora and fauna in the vicinity of the point of discharge.
**Description of data collection**	Physicochemical parameters were determined in the field. Samples for anions and cations were filtered using 0.45 µm CA filters and stored in 30 mL PE bottles. Cation samples were acidified with nitric acid. Samples for DOC (filtered, glass fibre filter 0.45 µm) and TOC were stored in 50 mL brown glass bottles and acidified with hydrochloric acid. Samples for fluorescence measurements were stored in 50 mL brown glass bottles. Silica samples were filtered using 0.45 µm CA filters and stored in 100 mL PE bottles.
**Data source location**	Water catchment area of the upper Schönmünz river, Black Forest National Park, southern Germany (48.55 - 48.59°N, 8.23 – 8.34°E)
**Data accessibility**	With the article
**Related research article**	Merk M, Goeppert N and Goldscheider N, "Processes Controlling Spatial and Temporal Dynamics of Spring Water Chemistry in the Black Forest National Park" (2020) *Science of The Total Environment, 723, Article: 137742, DOI: 10.1016/j.scitotenv.2020.137742*

**Value of the Data**

• The data can be used as a baseline for water chemistry of the springs and their respective ecosystems within the water catchment area.
• The dataset is a helpful and necessary supplement to ongoing investigations into the spring ecosystems, especially research of flora and fauna in the vicinity of the spring.
• Knowledge of the processes controlling spatial and temporal dynamics of spring water chemistry is important for creation of effective protection policies.

## Data Description

**Figure 1:**
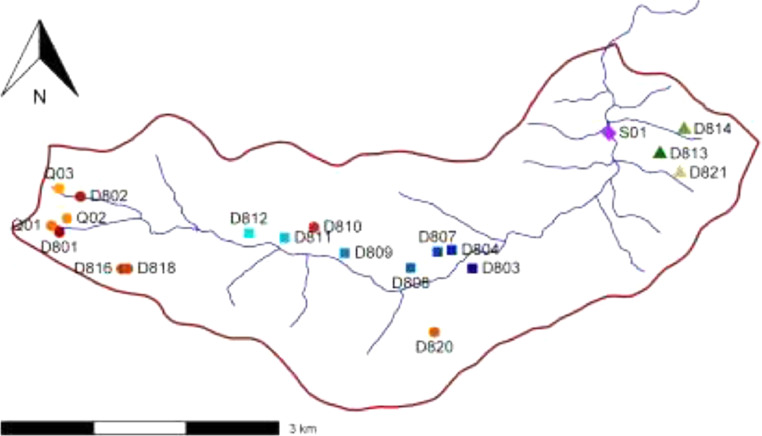
Map of the Schönmünz water catchment within the Black Forest National Park, southern Germany. Symbols indicate the sampling locations at the selected springs based on hydrogeology and topography (○, ☐, △) and the surface stream (◇).

Water samples were collected at 20 different locations within the study area during five different field campaigns. The dates of the sampling campaigns one to five are 25 May 2016 – 6 June 2016, 1 July 2016 – 8 July 2016, 5 September 2016 –8 September 2016, 2 November 2016 – 7 November 2016 and 8 May 2017 – 11 May 2017, respectively. The locations of the sampling points are listed in Tab. 1 and shown in Fig. 1. Because it was not possible to take samples at all of the numerous springs in the catchment area, a selection had to be made. Sampling points were chosen to include springs of differing elevation, geology and spring type in order to get a diverse overview of the hydrochemistry in the catchment area. Some sampling points were included in the sampling programme as they were already part of ongoing studies of the associated spring ecosystems. The final selection was based on stratigraphy, spring type as well as flora and fauna in the vicinity of the point of discharge. [1]

**Table 1:** Geographic coordinates of the selected sampling locations in the water catchment area of the upper Schönmünz River. (Coordinates in GK 3 reference frame, HW: “Hochwert”, northing; RW: “Rechtswert”, easting).

Spring	HW (m)	RW (m)	Elevation (m a.s.l.)	Group
D801	5381581	3443862	915	sandstone group
D802	5381967	3444088	911	sandstone group
D803	5381189	3448328	678	west granite group
D804	5381388	3448105	695	west granite group
D807	5381369	3447952	728	west granite group
D808	5381194	3447661	725	west granite group
D809	5381355	3446948	740	west granite group
D810	5381635	3446615	789	sandstone group
D811	5381520	3446299	734	west granite group
D812	5381568	3445913	738	west granite group
D813	5382427	3450357	695	east granite group
D814	5382692	3450617	688	east granite group
D816	5381186	3444539	898	sandstone group
D818	5381187	3444602	896	sandstone group
D820	5380502	3447918	829	sandstone group
D821	5382221	3450571	716	east granite group
Q01	5381653	3443774	958	sandstone group
Q02	5381731	3443941	920	sandstone group
Q03	5382053	3443859	955	sandstone group
S01	5382651	3449808	573	surface stream

Springs were grouped into the three clusters listed in Table 1, based on topography, geology and water chemistry. A summary of major ion chemistry is listed in Table 2.

**Table 2:** Summary of data by group and for all data.

			East granite group	Sandstone group	West granite group	Surface stream	All data
Elevation	m a.s.l.	min; max mean ± SD	688; 716 700 ± 15	789; 958 897 ± 55	678; 740 720 ± 24	573; 573 573 ± 0	573; 958 789 ± 112

Temperature	°C	min; max mean ± SD	7.03; 8.57 7.68 ± 0.45	4.83; 10.87 6.72 ± 1.18	5.93; 10.93 7.80 ± 0.82	4.90; 14.05 9.22 ± 3.29	4.83; 14.05 7.44 ± 1.42
pH	-	min; max mean ± SD	6.0; 6.90 6.48 ± 0.27	3.6; 5.12 4.33 ± 0.44	4.7; 6.61 5.74 ± 0.40	6.4; 7.42 6.95 ± 0.42	3.6; 7.42 5.38 ± 1.02
SEC	µS/cm	min; max mean ± SD	43.9; 117.3 76.5 ± 24.1	15.6; 61.0 29.0 ± 11.8	19.2; 36.8 26.9 ± 3.9	17.3; 34.7 25.3 ± 6.4	15.6; 117.3 35.3 ± 21.5
O_2_	%	min; max mean ± SD	83.9; 97.2 93.2 ± 3.2	85.9; 102.3 96.6 ± 3.7	63.8; 99.7 82.8 ± 10.7	100.9; 104.3 102.3 ± 1.4	63.8; 104.3 91.5 ± 9.7
EH	mV	min; max mean ± SD	522; 675 612 ± 41	566; 755 684 ± 47	494; 715 639 ± 63	505; 600 558 ± 32	494; 755 646 ± 63
HCO_3_^-^	meq/L	min; max mean ± SD	0.42; 1.10 0.69 ± 0.22	0.00; 0.05 0.01 ± 0.01	0.06; 0.28 0.14 ± 0.05	0.04; 0.26 0.17 ± 0.08	0.00; 1.10 0.17 ± 0.25

Ca^2+^	mg/L	min; max mean ± SD	3.93; 12.41 7.14 ± 2.49	0.02; 0.80 0.35 ± 0.21	1.17; 2.55 1.66 ± 0.33	1.42; 2.73 1.91 ± 0.50	0.02; 12.41 1.99 ± 2.52
Mg^2+^	mg/L	min; max mean ± SD	1.66; 6.33 3.48 ± 1.39	0.02; 0.21 0.12 ± 0.05	0.38; 1.35 0.67 ± 0.23	0.56; 1.15 0.84 ± 0.24	0.02; 6.33 0.89 ± 1.28
Na^+^	mg/L	min; max mean ± SD	0.54; 0.82 0.69 ± 0.08	0.32; 0.84 0.50 ± 0.10	0.44; 1.69 0.73 ± 0.34	0.66; 0.93 0.77 ± 0.11	0.32; 1.69 0.63 ± 0.24
K^+^	mg/L	min; max mean ± SD	0.97; 1.93 1.51 ± 0.27	0.07; 1.60 0.67 ± 0.37	0.58; 1.62 1.06 ± 0.25	0.56; 1.12 0.81 ± 0.23	0.07; 1.93 0.95 ± 0.42
Cl^-^	mg/L	min; max mean ± SD	0.90; 1.29 1.04 ± 0.11	0.37; 0.95 0.63 ± 0.13	0.70; 1.08 0.85 ± 0.07	0.77; 0.91 0.84 ± 0.05	0.37; 1.29 0.79 ± 0.18
NO_3_^-^	mg/L	min; max mean ± SD	0.85; 1.97 1.33 ± 0.39	0.25; 3.82 1.91 ± 0.98	0.58; 2.65 1.66 ± 0.52	0.39; 1.18 0.76 ± 0.29	0.25; 3.82 1.66 ± 0.79
SO_4_^2-^	mg/L	min; max mean ± SD	2.52; 5.87 3.52 ± 0.94	0.68; 2.61 1.38 ± 0.46	1.06; 4.39 2.44 ± 0.93	1.31; 1.99 1.73 ± 0.28	0.68; 5.87 2.12 ± 1.05

TDS	mg/L	min; max mean ± SD	38.31; 93.04 60.8 ± 18.1	2.74; 10.81 6.4 ± 2.3	9.82; 26.65 17.5 ± 3.6	8.64; 25.03 18.5 ± 6.0	2.74; 93.04 19.7 ± 20.0
TOC	mg/L	min; max mean ± SD	0.40; 2.00 0.7 ± 0.4	0.86; 27.53 11.6 ± 8.8	0.25; 9.01 1.6 ± 2.2	1.24; 11.43 5.8 ± 4.6	0.25; 27.53 5.9 ± 7.7
DOC	mg/L	min; max mean ± SD	0.32; 2.26 0.74 ± 0.54	0.76; 28.34 11.65 ± 8.86	0.18; 9.54 1.53 ± 2.29	1.20; 11.40 5.63 ± 4.43	0.18; 28.34 5.88 ± 7.77

Si	mg/L	min; max mean ± SD	2.03; 3.50 2.93 ± 0.36	0.98; 3.11 2.22 ± 0.50	1.83; 4.71 3.02 ± 0.68	2.02; 3.25 2.71 ± 0.43	0.98; 4.71 2.65 ± 0.66
Al	mg/L	min; max mean ± SD	0.00; 0.08 0.02 ± 0.03	0.00; 0.56 0.19 ± 0.19	0.00; 0.25 0.02 ± 0.05	0.00; 0.26 0.10 ± 0.11	0.00; 0.56 0.10 ± 0.16
Fe	mg/L	min; max mean ± SD	0.00; 0.06 0.03 ± 0.02	0.03; 0.39 0.13 ± 0.09	0.00; 0.14 0.04 ± 0.03	0.05; 0.15 0.09 ± 0.04	0.00; 0.39 0.08 ± 0.08

Coliforms	MPN/100 mL	min; max mean ± SD	1.0; 770.1 118.3 ± 232.9	1.0; 224.7 49.7 ± 49.8	<1; 2419.6 296.2 ± 638.0	115.3; 816.4 427.7 ± 260.0	<1; 2419.6 174.6 ± 416.9
*E. coli*	MPN/100 mL	min; max mean ± SD	<1; 13.2 1.4 ± 3.8	<1; 19.9 1.9 ± 3.9	<1; 17.3 1.1 ± 3.3	<1; 4.1 2.2 ± 1.7	<1; 19.9 1.5 ± 3.6

Enterococci	MPN/100 mL	min; max mean ± SD	<1; 5.1 0.68 ± 1.52	<1; 1.0 0.06 ± 0.25	<1; 2.0 0.32 ± 0.67	<1; 17.1 4.64 ± 7.13	<1; 17.1 0.55 ± 2.10
HIX	-	min; max mean ± SD	0.06; 1.44 0.42 ± 0.41	0.04; 0.08 0.06 ± 0.01	0.04; 1.36 0.38 ± 0.40	0.04; 0.10 0.07 ± 0.02	0.04; 1.44 0.23 ± 0.33
BIX	-	min; max mean ± SD	0.53; 0.96 0.68 ± 0.14	0.38; 0.50 0.43 ± 0.03	0.40; 3.20 0.73 ± 0.54	0.40; 0.44 0.42 ± 0.02	0.38; 3.20 0.58 ± 0.36
Abs(254)	-	min; max mean ± SD	-0.01; 0.08 0.02 ± 0.02	0.04; 1.38 0.62 ± 0.45	-0.01; 0.44 0.07 ± 0.12	0.05; 0.60 0.33 ± 0.24	-0.01; 1.38 0.31 ± 0.41

Absorbance data files (*.abs) (Appendix A, Absorbance) are comma separated files containing measured absorbance spectra. Files are named by sampling station, sampling campaign and sampling date, separated by an underscore. Data columns in the files are: Wavelength (nm); I1 (µA) Abs Detector Raw; I1 dark (µA) Dark Offset for Abs Detector; R1 (µA) Ref Detector raw; R1dark (µA) Dark Offset for Ref Detector, XCorrect (-) Linear interp; I1c (µA Dark subtracted Abs Detector); R1c (µA) Corrected Ref Detector, I1c/R1c (µA/µA) Corrected Intensity; Abs (OD) –Log(T); Percent T (T*100) % Transmittance.

Fluorescence data files (*.csv) (Appendix A, Fluorescence) are comma separated files containing measured fluorescence excitation-emission matrices (EEM) in counts per second, as measured by the device. Files are named by sampling station, sampling campaign and sampling date, separated by an underscore. The first data column contains the emission wavelengths (nm) and the first data row contains the excitation wavelengths (nm).

The shapefile (Appendix A, Locations) contains the locations of all sampling stations within the water catchment area. Coordinate reference system is the German DHDN GK3, based on the Bessel 1984 spheroid.

The spreadsheet in the appendix (Appendix A, Table S1) contains individual values of measured data: Location, Date, Time, campaign number, specific electrical conductivity (SEC), pH, O_2_, HCO_3_^-^ , measured redox potential, corrected standard redox potential (E_H_), Temperature (T), concentrations of: Cl^-^, SO_4_^2-^, NO_3_^-^, PO_4_^3-^, Na^+^, K^+^ , Mg^2+^, Ca^2+^, Fe_tot_, aluminium (Al),silica (Si), total dissolved solids (TDS), acidification quotient (AQ), most probable numbers (MPN/100 mL) for Coliforms, *E. coli* and Enterococci, concentrations of total organic carbon (TOC) and dissolved organic carbon (DOC), biological index (BIX) [2], humification index (HIX) [3] and spectral absorption coefficients (SAK) at wavelength of 210, 254, 280, 436 and 545 nm as well as discharge and assignment to geologic group according to [1].

## Experimental Design, Materials, and Methods

### Field sampling

Physicochemical parameters were determined in the field. Temperature, specific electrical conductivity (SEC), dissolved oxygen (DO), the redox potential and pH were taken using a WTW Multi 3430 and the respective probes.

A digital Sentix 82 probe was used during the first sampling campaign to measure pH. As this probe is not well suited for waters with low mineralization, these measurements did not pass quality control measures and could not be included in this dataset or used in data analysis. During the second sampling campaign, an analog Sentix HW probe in combination with an analog TetraCon 325 for temperature compensation and a WTW Multi 340i was used. A digital Sentix HW-T 900P probe was used during the other sampling campaigns, as this type is especially suitable for low mineralization waters.

The probe used for measurement of SEC was a TetraCon 925.

An optical probe of type FDO 925 was used to determine DO.

Redox potentials were not determined during the first sampling campaign. In campaigns two through five, a Sentix ORP-T 900 was used. Equilibration times of at least 20 minutes were observed before readings of redox were taken, as these probes are known to have long equilibration times. Measured values were converted into standard hydrogen electrode potentials by adding a temperature dependent reference voltage according to the documentation by the manufacturer. All probes – except the redox probe – had an integrated temperature sensor. Measured temperatures differed slightly between probes. For correction of redox potentials and data analysis, the mean was used.

Alkalinity was determined by titration of 100 mL of sample water with a solution of 25 mmol/L hydrochloric acid in the field.

Samples for anion and cation determination were filtered using 0.45 µm CA filters and stored in 30 mL PE bottles. Cation samples were acidified with 50 µL nitric acid (65%, suprapur). No chemical was added to samples for determination of anions. Silica samples were filtered using the same 0.45 µm CA filters and stored in 100 mL PE bottles.

Samples for determination of DOC were filtered using glass fibre filters with a mean pore size of 0.45 µm. These filters were heat treated at 450 °C for a minimum of 4 h to remove residual carbon from within the filter. Brown glass bottles with a volume of 50 mL were used for samples designated for DOC, TOC and fluorescence analysis. Samples for TOC were taken unfiltered. For inhibition of bacterial and algae growth and removal of inorganic carbon, pH of DOC and TOC samples was lowered using 250 mL of hydrochloric acid (37%, suprapur).

Samples for fluorescence spectroscopy were taken unfiltered and stored without addition of acid or any other stabilizing agents. No samples for TOC and fluorescence analysis were taken during the second sampling campaign.

All sample containers and sample taking equipment was rinsed several times and filters thoroughly flushed with sample water before taking the actual sample. Sample bottles were stored in a Styrofoam box, using cool packs as necessary and transported to the lab daily. All samples were subsequently stored in the dark at +8 °C and usually analysed within one week.

For microbial analysis of coliforms, *E. coli* and enterococci, two sterile PS-bottles (Idexx) were filled up to the 100 mL mark.

Discharge measurements were done using two different methods. The method used is listed next to discharge measurements in the supplemented data. Volumetric measurements were mainly done at the springs. Discharge from the spring was collected in a plastic bag that was pressed to the streambed. The time was measured using a stopwatch and the amount of water that was collected was subsequently measured using a measuring cylinder. In cases where the complete flow could not be retrieved, the proportion of the water that was missed was approximated. These measurements are marked as estimates.

The salt-dilution method [4] was used for discharge measurements of the surface stream.

### Analysis

Element concentrations of Mg^2+^, K^+^ and Na^+^ were determined using ion chromatography (ICS-1100, Thermo Fisher Scientific). Ca^2+^ concentrations were determined by flame AAS (Perkin-Elmer 3030 B). Concentrations of Cl^-^, SO_4_^2-^ and NO_3_^-^ were similarly determined using ion chromatography (ICS-2100, Thermo Fisher Scientific). TOC and DOC concentrations were determined by oxidative combustion at 850 °C and subsequent determination of carbon dioxide (Vario TOC cube, Elementar Analysesysteme GmbH).

Simultaneous measurement of absorbance spectra and fluorescence excitation-emission matrices (EEM) were done by fluorescence spectroscopy (Aqualog, Horiba Scientific). An integration time of two seconds was used for excitation wavelengths from 200 nm to 600 nm in steps of 3 nm. EEMs were corrected for inner filter effects and Raman scattering was removed in the software provided by the manufacturer. Measured intensities were used directly for further analysis without calibrating to any standard. Measured absorbance spectra have a resolution of 3 nm starting at 201 nm and ending at 600 nm, requiring absorbance at a specific wavelength to be interpolated linearly between its neighbours.

Dissolved silica was measured using the classical molybdenum blue method. Ammonium molybdate is added to the sample to form a complex of dissolved silica and molybdic acid. This complex is reduced by stannous chloride to molybdenum blue. Absorption measurements were done at 810 nm using a lab photometer (Photolab 6600 UV-Vis, WTW). Five mL of sample were diluted by 15 mL of distilled water to reduce equilibration times of the complex formation. Measured absorbance values were converted into Si-concentrations by use of calibration standards (prepared daily) and linear regression.

Aluminium was determined using a photometer and a sample kit (Spectroquant aluminum test, Merck). Al dissolved by organic complexation with humic substances or other organic molecules (Al_(org)_) and Al that is dissolved inorganically as Al^3+^ or different Al-hydroxides (Al_(inorg)_) have not been measured separately.

Total iron (Fe^2+/3+^) was determined using a test kit (Aquaquant iron test, Merck).

Microbiological data was acquired by adding substrate powder directly to the designated 100 mL samples and shaking until dissolved. For determination of coliforms and *E. coli*, Colisure (Idexx) [5] was used. For determination of enterococci, Enterolert (Idexx) [6] was used.

After adding a substrate powder, each sample was transferred to a Quanti-Tray/2000 within 12 hours after sampling, sealed and incubated for 24 hours at the specified temperature (Colisure: 35 °C, Enterolert: 42 °C). The number of positive indentations was then counted and the most probable number (MPN/100mL) calculated from the supplied conversion table.

## Declaration of Competing Interest

Financial support for this study was provided by the Black Forest National Park.

## Appendix Supplementary materials

Image, application 1

Image, application 2

Image, application 3

Image, application 4

Image, application 5
